# Global transcriptome analysis reveals circadian regulation of key pathways in plant growth and development

**DOI:** 10.1186/gb-2008-9-8-r130

**Published:** 2008-08-18

**Authors:** Michael F Covington, Julin N Maloof, Marty Straume, Steve A Kay, Stacey L Harmer

**Affiliations:** 1 Department of Plant Biology, College of Biological Sciences, One Shields Avenue, University of California, Davis, California 95616, USA; 2 Present address: Department of Biochemistry and Cell Biology, Rice University, Main Street, Houston, Texas 77005, USA; 3 Center for Biomathematical Technology, Box 800735, University of Virginia Health Sciences System, Charlottesville, Virginia 22908, USA; 4 Present address: Customized Online Biomathematical Research Applications, Glenaire Drive, Charlottesville, Virginia 22901, USA; 5 Department of Biochemistry, The Scripps Research Institute, North Torrey Pines Road, La Jolla, California 92037, USA; 6 Present address: Section of Cell and Developmental Biology, University of California at San Diego, Gilman Drive, La Jolla, California 92093, USA

## Abstract

**Background:**

As nonmotile organisms, plants must rapidly adapt to ever-changing environmental conditions, including those caused by daily light/dark cycles. One important mechanism for anticipating and preparing for such predictable changes is the circadian clock. Nearly all organisms have circadian oscillators that, when they are in phase with the Earth's rotation, provide a competitive advantage. In order to understand how circadian clocks benefit plants, it is necessary to identify the pathways and processes that are clock controlled.

**Results:**

We have integrated information from multiple circadian microarray experiments performed on *Arabidopsis thaliana *in order to better estimate the fraction of the plant transcriptome that is circadian regulated. Analyzing the promoters of clock-controlled genes, we identified circadian clock regulatory elements correlated with phase-specific transcript accumulation. We have also identified several physiological pathways enriched for clock-regulated changes in transcript abundance, suggesting they may be modulated by the circadian clock.

**Conclusion:**

Our analysis suggests that transcript abundance of roughly one-third of expressed *A. thaliana *genes is circadian regulated. We found four promoter elements, enriched in the promoters of genes with four discrete phases, which may contribute to the time-of-day specific changes in the transcript abundance of these genes. Clock-regulated genes are over-represented among all of the classical plant hormone and multiple stress response pathways, suggesting that all of these pathways are influenced by the circadian clock. Further exploration of the links between the clock and these pathways will lead to a better understanding of how the circadian clock affects plant growth and leads to improved fitness.

## Abstract 2

Transcript abundance of roughly a third of expressed *Arabidopsis thaliana* genes is circadian-regulated.

## Background

Harsh environmental extremes often accompany the daily light-dark cycle. In nearly every organism studied an endogenous time keeping mechanism has evolved that enables anticipation of these predictable changes [1]. This is especially critical for sessile organisms such as plants. The circadian clock produces self-sustained rhythms with a period length of approximately 24 hours. To keep these rhythms in proper alignment with the day-night cycle, the clock is set or entrained by environmental timing cues such as changes in light or temperature. This is important because a functional clock can only provide an organism with a competitive advantage when it is correctly matched to the external environment [2,3].

Although this advantage has been demonstrated for both phytoplankton and higher plants, the mechanistic link between the circadian clock and increased fitness remains unclear. Understanding how clocks confer an adaptive advantage requires a thorough knowledge of circadian-regulated pathways and processes. Fortunately, several microarray experiments have been performed to identify the circadian transcriptome of the model plant system *Arabidopsis *[4-8]. These studies have shown that a substantial portion of the plant genome is clock controlled, with transcript levels of different genes showing peak accumulation at all times, or phases, of the circadian cycle. We and others refer to genes with rhythmic regulation of transcript abundance as 'clock-regulated'; this may reflect circadian regulation of promoter activity and/or mRNA stability.

This raises another major question in circadian biology; how does the central clock mechanism control the vast array of circadian outputs and phase them to the appropriate time of day? Although the circadian clocks of higher plants, animals, and fungi consist of interlocking transcriptional feedback loops, the individual components vary [9-11]. In plants, one of these loops involves the reciprocal regulation of CCA1 (circadian clock associated 1) and TOC1 (timing of CAB expression 1), which have morning and evening phases of peak expression, respectively [12]. Whereas TOC1 promotes *CCA1 *expression, the myb-related transcription factor CCA1 represses *TOC1 *expression upon binding to a circadian clock regulatory element (CCRE) in the *TOC1 *promoter [12,13]. This CCRE, called the evening element (EE), is over-represented in the promoters of evening expressed circadian genes, and when multimerized it drives evening-phased circadian regulation of a reporter gene [14]. The EE is one of the few CCREs that have been characterized [4,8,14,15]. Several more CCREs, however, are likely required to generate the enormous diversity observed in phases of transcript accumulation of clock-regulated genes.

Here we suggest that the abundance of as many as one-third of expressed transcripts in *Arabidopsis *is circadian regulated; we use data from multiple circadian microarray experiments to discover known and potential circadian clock regulatory elements; and we identify new circadian-enriched pathways that may help to explain the physiological importance of the clock. These findings may help explain how clock outputs are regulated so that they occur at the appropriate time of day, a central function of the circadian clock [2]. In addition, the enrichment of clock-regulated genes among many phytohormone- and stress-response pathways suggests that the circadian system modulates plant responses to most hormones and stresses, probably contributing to the adaptive advantage provided by a properly phased clock [2]. These findings suggest the clock plays fundamental roles in nearly all aspects of plant growth and development, as well as in plant environment interactions.

## Results and discussion

### Comparison of circadian microarray datasets

**Figure 1:**
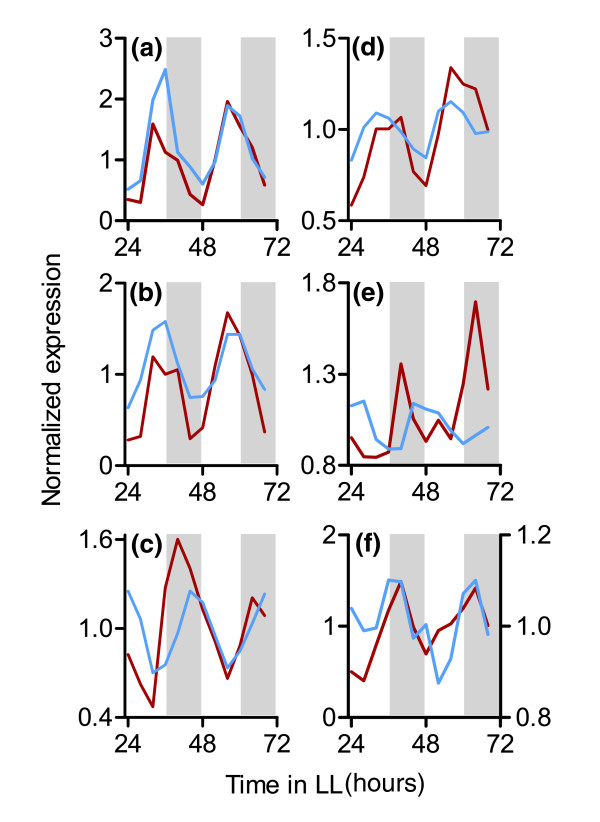
Validation of circadian microarray data by RT-PCR. Expression data from two independent time courses (blue = microarray; red = RT-PCR) for randomly chosen **(a-c) **high amplitude (At1g06460, At1g69830, and At5g12110) and **(e-f) **low amplitude (At3g22970, At1g45688, and At3g04760) circadian-regulated genes. Amplitude classification is based on microarray analysis [7]. For panel f, RT-PCR and microarray data are plotted on the left and right y-axes, respectively. White and gray shading represent subjective day and night, respectively.

Rhythmic control of gene expression is an important function of the circadian system; however, genome-wide microarray studies performed on *Arabidopsis *have yielded varying estimates of the fraction and identity of genes that are clock regulated. We recently found that the abundance of 10.4% ('Covington dataset') of expressed transcripts is circadian regulated in light-grown *Arabidopsis *seedlings [7]. To evaluate experimentally the prevalence of false positives in this dataset, we randomly chose six genes identified as circadian but with predicted high and low amplitudes. We then assessed transcript abundance of these genes by RT-PCR using samples derived from an independent circadian time course. We found that all of the genes tested were circadian regulated (Figure 1), suggesting that the false-positive rate for the Covington dataset, as previously analyzed, is likely to be low. Indeed, analysis of simulated data has led to the conclusion that COSOPT (the algorithm we used to detect rhythmic changes in transcript abundance) minimizes false positives at the expense of increased false negatives [16]. Our analysis of a simulated dataset (random values with a mean of 0 and a standard deviation of 1) using the same parameters as the original Covington analysis indicates a false-positive rate of 1.6%, which corresponds to a false-discovery rate of 9.6%.

**Figure 2:**
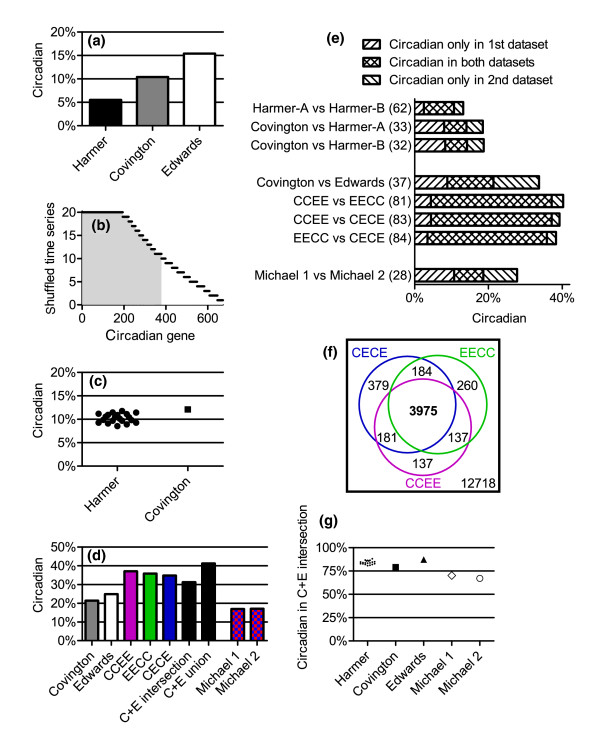
Comparison of three circadian microarray datasets. The power to detect circadian genes is greatly increased when independent datasets are combined. **(a) **The degree of circadian regulation of the *Arabidopsis *genome as originally reported in different studies [4,6,7]. **(b) **The number of unique unreplicated time series (generated by random shuffling of Harmer technical replicates) that identifies each of the circadian-regulated genes found in at least one shuffled time series. The shaded portion indicates the genes that are found to be circadian in a majority of the time series. **(c) **The shuffled Harmer datasets were analyzed according to the parameters originally used for the Covington dataset; only genes common to the two microarray platforms were considered. **(d) **The Covington dataset was reanalyzed according to the parameters originally used for the Edwards dataset, with the exception that only genes expressed in both datasets were evaluated. Also shown are the results of the analysis of the combined Covington and Edwards datasets, as well as the Michael datasets. For the individual and combined Covington plus Edwards datasets, only genes that are expressed in both of the individual data sets are considered. **(e) **The unions and intersections of sets of genes determined to be circadian expressed by the different datasets. Harmer-A and Harmer-B represent the two of the 20 shuffled datasets with the degree of circadian regulation closest to the 50th percentile. The percent overlap for each pair is shown in parentheses. **(f) **There is substantial overlap in the identity of circadian regulated genes (shown as numbers within Venn diagram circles) found by the three combined Covington plus Edwards datasets. The number in the lower right represents the number of genes that are expressed in both the Covington and Edwards datasets. **(g) **Collections of circadian genes identified in different datasets share substantial identity with the circadian genes found by each of the three combined Covington and Edwards datasets.

Studies using very similar entrainment and growth conditions have resulted in reports that expression of 5.5% ('Harmer dataset') to 15.4% ('Edwards dataset') of genes is circadian regulated [4,6] (Figure 2a). Many factors could lead to these discrepancies, including differences in experimental and analytical techniques (Table 1). To compare the datasets properly, we minimized these differences by applying standardized analysis procedures to all three experiments. Because the Harmer dataset has two technical replicates per time point whereas the Covington and Edwards datasets each have one array per time point, we reanalyzed the Harmer data using only one microarray per time point. We created 20 different unreplicated time course series in this manner, using different combinations of arrays for each randomly 'shuffled' time course. Because all other factors were constant, comparison of cycling genes in these time series allows us to assess the variability associated with microarray hybridization and processing. Using COSOPT with the stringency threshold (pMMC-β, a multiple-measures-corrected significance probability for the rhythmic amplitude parameter, which is based upon analysis of randomized data) set to 0.05 [7], we found that the fraction of clock-regulated genes in these series were similar, ranging from 9% to 12%. However, the mean overlap of genes found to be circadian regulated in both 'shuffled' time courses when any two lists are compared is only 54% (number of circadian genes in common/number of circadian genes total). Although 29% of the genes found to be circadian regulated by any of the 'shuffled' time series are identified as circadian in every time series, only 56% are identified as circadian in at least 11 of the 20 time series (Figure 2b). These results suggest that variability in microarray processing, even within the same facility, can contribute greatly to variation between microarray experiments.

**Table 1:** Experimental differences in original circadian microarray analyses

Publication	% Circadian	Number of time points	Light intensity (μmol/m^2 ^per second)	Microarray platform	Technical replicates	Low-level analysis	Circadian detection algorithm	Presence cut-off
Harmer and coworkers [4]	5.5	12	60	Affymetrix *Arabidopsis *Genome	2	Affymetrix MAS 4.0	CORRCOS	None
Edwards and coworkers [6]	15.4	13	60 to 65	Affymetrix *Arabidopsis *ATH1	N/A	GC-RMA	COSOPT (less stringent)	None
Covington and Harmer [7]	10.4	12	120	Affymetrix *Arabidopsis *ATH1	N/A	dChip	COSOPT (more stringent)	Genes present in ≥ 4 of 12 samples

We next compared the degree of circadian regulation found in the Harmer and Covington datasets when the same analytical techniques are used. Comparing only genes found on both of the array platforms used in these experiments, the degree of circadian regulation in the Harmer and Covington datasets is quite similar (Figure 2c). When the Covington and Edwards datasets are analyzed using the same method used in the original Edwards analysis [6], the percentage of genes designated as clock regulated in the two experiments also becomes much more similar (Figure 2d). However, the degree of overlap between the genes defined as clock regulated in both the Harmer and Covington datasets or Edwards and Covington datasets is limited: about 33% and 37%, respectively (Figure 2e).

We suspected that genes identified as circadian regulated in both the Covington and Edwards microarray studies have high amplitude rhythms, whereas genes with low amplitude rhythms tended to be identified in only one of the studies. As predicted, we found a strikingly significant difference (*P *= 1.7 × 10^-106^) between the relative amplitude of rhythmic genes identified by both datasets (0.21) and that of rhythmic genes identified only by the Covington dataset (0.12). This, together with our analysis of the Harmer dataset, suggested that identification of clock-regulated genes might be limited by technical issues and would benefit from increased sample numbers.

Because the Edwards and Covington experimental procedures were very similar, we reasoned that we might gain power by analyzing the 25 microarrays from these two experiments as a single time series. After normalizing the expression values for each probe set to its median for each dataset, we combined the two experiments in three ways: by interweaving these datasets to generate a 2-hour resolution time course spanning two days ('CECE' dataset); by appending the Edwards series after the Covington series to generate a 4-hour resolution time course over four days ('CCEE' dataset); and by appending the Covington series after the Edwards series to generate a different 4-day time course ('EECC' dataset; see Additional data file 1).

All three time courses were analyzed in accordance with the parameters used in the original Edwards analysis [6]. In each case the abundance of 35% to 37% of expressed transcripts was found to be clock-regulated (Figure 2d). These three gene lists were remarkably consistent, with all two-way comparisons of these gene lists having 81% to 84% overlap (Figure 2e) and the intersection of all three lists being 76% of the union (Figure 2f). This group of 3,975 predicted circadian-regulated genes ('C+E intersection') at the intersection of the combined Covington and Edwards datasets contains almost all of the circadian genes found by analysis of the individual Covington and Edwards datasets (79% and 87%, respectively) as well as by the 'shuffled' Harmer time courses (81% to 88%; Figure 2g). Analysis of simulated data indicates that the strategy to identify the circadian-regulated genes in the C+E intersection has a false-positive rate of 1.1% and a false-discovery rate of 2.8%, which are much better than that for a single time course of 12 time points analyzed with the more stringent parameters used in the original Covington analysis (1.6% and 9.6%, respectively).

Two additional circadian microarray experiments ('Michael datasets') were recently performed using *Arabidopsis *seedlings and the same platform as the Covington and Edwards datasets [8]. Subjecting the Michael datasets to analysis with our parameters reveals 17% circadian regulation in each dataset (Figure 2d) with limited overlap of circadian genes (Figure 2e). Seedlings harvested for the Michael datasets were grown differently than those used for the Covington, Edwards, and Harmer datasets. These differences included growth on media lacking sucrose and entrainment by daily changes in temperature (either in constant light ('Michael 1' dataset) or in combination with light/dark cycles ('Michael 2' dataset). Remarkably, even despite these differences, more than two-thirds of the circadian genes identified in our analysis of the Michael datasets are also found in the C+E intersection (Figure 2g).

A recent comparison of five independent microarray studies to identify circadian-regulated genes in *Drosophila *[17] demonstrated that differences in circadian detection algorithms as well as laboratory-dependent differences both have significant impacts on the overlap of lists of circadian-regulated genes. Even when they were reanalyzed in a uniform manner, the maximum observed overlap between lists of circadian-regulated genes from any two *Drosophila *datasets was only 24%, with an average overlap of 11%. The extensive overlap of cycling genes found between the C+E intersection and each of the individual datasets (Harmer, Covington, Edwards, and the two Michael datasets) suggests that a major limitation for detecting clock-regulated genes in circadian microarray experiments is not laboratory dependent or biological variation, but rather technical issues that can be alleviated by increasing the number of time points. This can be accomplished by increasing the duration of the time course, the sampling frequency during the time course, or the degree of biological replication of samples. The first two approaches provide more biological information and thus appear to be preferable to the third. In order to minimize developmental effects and the damping of rhythms that often occurs during free running conditions, we recommend circadian time courses with increased sampling frequency rather than increased duration.

Given the impressive overlap between the genes designated as clock regulated when the Covington and Edwards datasets are either appended end-to-end or interwoven (Figure 2e, f), it appears reasonable to conclude that between 31% and 41% of expressed genes (representing the intersection and the union of the cyclers found in these datasets, respectively) are under circadian regulation (Figure 2f). This is consistent with an estimate of 36% of genes being circadian regulated based on a luciferase-based enhancer-trapping approach [18]. For a summary of the genes that are expressed and circadian in the individual and combined datasets, see Additional data file 2.

### Genome organization of circadian-regulated genes

Co-expressed genes have been shown to occur in clusters throughout the *Arabidopsis *genome [19,20]. Similar patterns of genome organization have also been observed in animals and fungi [21,22]. To determine whether genome organization plays an important role in circadian regulation of gene expression, we used three computational approaches to look for patterns in genome location of clock-regulated genes. We calculated the Pearson product-moment correlation coefficient, the fraction of clustered clock-regulated genes, and the mean pMMC-β value (a significance measure for circadian rhythmicity) in a sliding window across multiple genes to test whether circadian-regulated genes are co-localized in the *Arabidopsis *genome.

**Figure 3:**
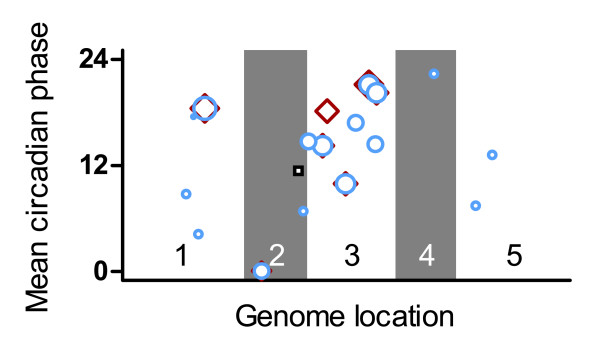
Identification of local clusters of circadian-regulated genes. Genome location (x-axis) and mean circadian phase (y-axis) are shown for clusters of circadian-regulated genes. Eighteen clusters were identified based on the proportion of circadian-regulated genes (red diamonds), the mean pMMC-β value (blue circles), or the mean combinatorial pair-wise Pearson correlation coefficient (black squares) in a sliding window of 2, 5, or 10 genes. The number of circadian-regulated genes within each cluster (ranging from one to six genes) is represented by the size of the corresponding symbol. The individual chromosomes are indicated by shading and numbers.

Combining the results from all three cluster discovery methods, we found only 18 unique circadian clusters. These represent only 63 of the 3,975 circadian-regulated genes identified in the C+E intersection (Figure 3). Functionally related genes are often co-expressed [20], suggesting that some of the above clusters might consist of genes that act in the same pathways. Consistent with this possibility, five out of the 18 circadian clusters contain multiple members of specific gene families. This co-expression may therefore be due to conserved regulatory regions resulting from gene duplications. The very limited clustering of clock-regulated genes suggests that circadian regulation of chromatin organization [13] does not play an important role in the regulated expression of adjacent genes.

### Analysis of circadian clock regulatory elements

**Figure 4:**
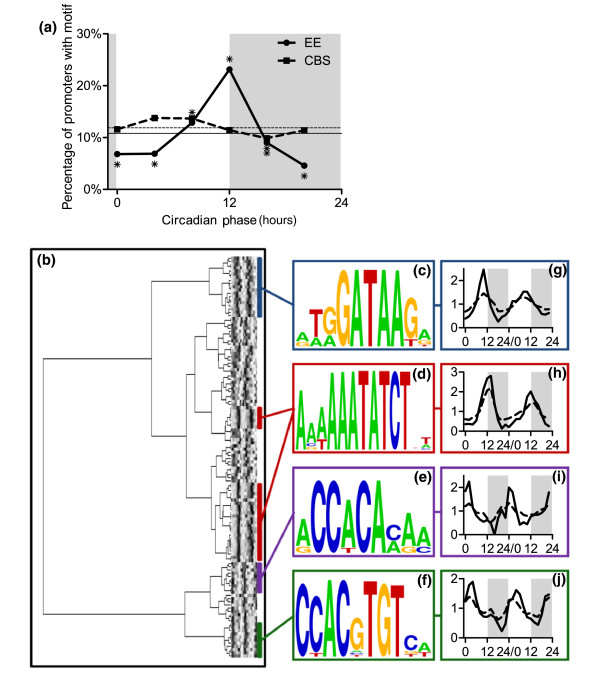
Analysis and identification of regulatory elements in the promoters of circadian-expressed genes. **(a) **Frequency of the evening element (EE) and CCA1-binding site (CBS) motifs in the promoters of circadian-regulated genes classified by phase of peak expression. Asterisks indicate phases during which the frequency of promoters containing the motif is significantly different from that of all circadian promoters. Asterisks are placed above the data point to indicate over-representation of the motif and below to indicate under-representation. Both the EE and the CBS are under-represented in promoters of genes with peak expression at circadian time 16. The horizontal lines indicate frequency of the motifs (solid line = EE; dashed line = CBS) in the promoters of all circadian-regulated genes. **(b) **Tree of putative circadian clock regulatory elements (CCREs) clustered based on sequence similarity is plotted adjacent to a heat map that represents the frequency of each motif in phase-specific subsets of the promoters of genes determined to be circadian regulated in the original analyses of the Covington (left half of heat map) and Edwards (right half of heat map) datasets [6,7]. In the heat map, dark and light shading represent high and low frequency, respectively. **(c-f) **Consensus sequences depicted as sequence logos are shown for select clades. **(g-j) **The phase-specific frequencies of the consensus sequences are plotted in a similar manner as in panel a, except that frequency data are shown for both the Covington (first 24 hours) and Edwards (second 24 hours) datasets and is normalized to the frequency of the sequence in the promoters of all circadian genes. The mean phase-specific frequencies for all the motifs in a clade are shown as dashed lines. For panels a and g to j, white and gray shading represent subjective day and night, respectively.

The clock component CCA1 represses *TOC1 *expression by binding directly to its promoter [12,13]. This promoter region contains an EE (AAAATATCT), a CCRE required for the evening-phased expression of *TOC1*, and other genes [4,12,23]. CCA1 also binds a highly related motif called the CCA1-binding site (CBS; AAAAAATCT) [24]. Both the EE and CBS are significantly over-represented in the promoters of circadian-regulated genes found in the C+E intersection (Figure 4a). The CBS has been suggested to be a phase-specific CCRE present in the promoters of dawn-phased genes [23]; however, a multimerized version of the CBS drives luciferase expression with the same evening-phased expression as an EE multimer [14].

To evaluate the biological relevance of the CBS, we examined the phase distributions of circadian-regulated genes containing the CBS and, as a control, the related EE motif. EEs are over-represented in the promoters of evening-phased genes and are under-represented in the promoters of genes with transcripts that accumulate at any other time of day, as previously reported (Figure 4a) [4,8]. In contrast, the CBS is only under-represented in one and is not over-represented in any phase groups (Figure 4a), which suggests that the CBS is not involved in phase-specific transcript accumulation. It may be that both the *in vitro *binding of CCA1 to the CBS and the evening-phased circadian regulation conferred by the multimerized CBS are artifacts caused by the high similarity between the CBS and the EE.

Only two other CCREs have been demonstrated to control phase-specific expression; when multimerized, the morning element (ME; AACCACGAAAAT) confers dawn-phased expression and the protein box element (PBX; ATGGGCC) confers midnight-phased expression on a luciferase reporter gene [8,14]. Therefore, the question remains, how is the observed diverse array of circadian phases of transcript abundance generated? To identify motifs that are important for time-of-day-specific circadian expression, we developed a multipronged promoter motif discovery and validation approach (described in Materials and methods, see below). We reduced the number of possible CCREs with the stringent requirement that each candidate motif exhibit phase-specific over-representation among genes classified as circadian in both the Covington and Edwards datasets. These candidate CCREs were then clustered based on their sequence similarity, leading to the identification of clades of related motifs (Figure 4b). When we calculated the frequency of each motif in the promoters of circadian-regulated genes, we found that most of the clades exhibit the same phase of peak transcript abundance in both the Covington and the Edwards datasets, validating our approach (see heat map in Figure 4b). The clusters with the greatest degree of phase consolidation contain genes with transcript abundance peaking during subjective dawn (Figure 4e), early day (Figure 4f), late day (Figure 4c), and subjective dusk (Figure 4d). As expected, the frequency distribution data for these consensus sequences correlate with the mean phase-specific frequencies of all motifs in the indicated clades (Figure 4g-j).

The putative CCREs that we identified are related to motifs recently found by others to be enriched in the promoters of circadian genes [4,8,14,15]. The CCACA motif that we found to be enriched in the promoters of dawn-phased genes (Figure 4e) is almost identical to the ME computationally defined by Michael and coworkers [8] and similar to the ME found by Harmer and Kay [14] to confer dawn-phased rhythms on a reporter gene. Similarly, the early day-phased motif shown in Figure 4f contains a G-box sequence, which Michael and coworkers [8] found to be enriched in dawn-phased genes. The late day-phased motif (Figure 4c) contains a GATA core element, which is also found within the longer EE motif (Figure 4d). Interestingly, the GATA cluster has a slightly earlier phase than the EE cluster, suggesting that specific flanking sequences might modify the phase conferred by a CCRE. Indeed, we previously showed that placing a ME adjacent to an EE in the promoter of a reporter gene results in an advanced phase of expression relative to an EE alone [14]. Michael and coworkers [8] also found that GATA motifs are enriched in the promoters of genes with an afternoon phase of transcript accumulation.

Despite using different analytical strategies and gene lists, we and Michael and coworkers [8] found many of the same motifs to show phase-specific enrichment. This strongly suggests that the field has now identified at least four major motifs important for clock-regulated transcript accumulation at multiple phases during the subjective day and night. There may be other important CCREs yet to be discovered, because our analysis [14] did not identify the PBX motif found by Michael and coworkers [8].

It will next be critical to test whether the GATA and G-box motifs do confer different day-phased rhythms of transcript accumulation and to determine whether different combinations of the four known CCREs in the promoters of circadian genes are sufficient to confer every phase of circadian transcript accumulation. Identification of the transcription factors that bind to these CCREs will provide insight into the circuitry of the circadian clock and the regulatory network between the clock and its outputs.

### Circadian transcription factors

To begin to define this regulatory network, we next wished to identify transcription factors found to be clock regulated in the C+E intersection. Only 732 of the 1,690 genes with the GOslim annotation [25] 'transcription factor activity' are detectably expressed in the C+E intersection, perhaps reflecting specialized functions of many transcription factors in nonseedling tissues. Of these 732 genes, we found 247 (33.7%) - from a variety of families - to be circadian regulated. Although this degree of circadian regulation is no higher than would be expected by chance, seven transcription factor families exhibit a significant circadian enrichment: Constans (CO)-like, Myb-related, basic leucine zipper (bZIP), multiprotein bridging factor 1 (MBF1), barley B recombinant-basic pentacysteine 1 (BBR-BPC), tubby-like protein (TLP), and teosinte branched1/cycloidia/PCF (TCP).

Links to the circadian clock were previously described for the first three families [10,26-32] but not for the others. A role for plant homologs of MBF1 in defense responses to pathogens has been suggested [33], whereas members of the BBR-BPC, TLP, and TCP families have been implicated in multiple aspects of development control [34-37]. For the TCP transcription factors, this includes cell growth and proliferation, organ shape and border delimitation, and shoot branching [37]. Perturbation of expression of clock-regulated *TCP *genes causes phenotypes often found in clock mutants, such as late flowering and elongated hypocotyls [38], suggesting these plants may have impaired circadian function.

### Identification of pathways with an under- or over-representation of circadian-regulated genes

In order to understand the physiological relevance of the circadian system and how a functional clock can confer a competitive advantage [2], we must know which pathways and processes are controlled by the clock. We therefore identified functionally-related gene groups with either more or fewer circadian-regulated genes than expected by chance. Many core processes had significantly fewer than expected oscillatory transcripts, including the following: RNA processing; DNA synthesis and chromatin structure; protein synthesis, secretion, and ubiquitin-mediated degradation; G-protein-mediated signaling; and cell cycle. It may be that these processes are not clock regulated because they must occur during all times during the day/night cycle. On the other hand, transcript abundance of these genes may only be clock regulated in a subset of tissue types; if this is the case, then we might not detect circadian regulation given the whole-plant sampling performed in published microarray studies. Finally, these pathways might be influenced by the circadian clock either via clock-controlled transcription of one or a few key regulators or via circadian influence on post-transcriptional mechanisms such as protein degradation or phosphorylation [39,40].

### Circadian regulation of isoprenoid biosynthetic pathways and ABA biosynthetic genes

**Figure 5:**
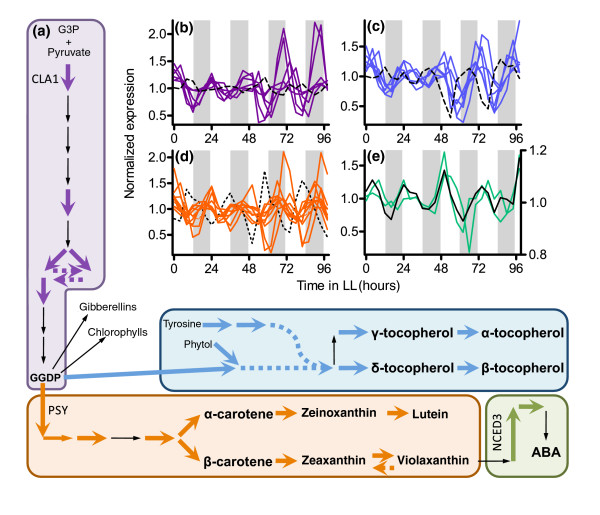
Circadian co-regulation of metabolic pathways. **(a) **Metabolic pathways for the production of the key intermediate geranylgeranyl diphosphate (GGDP), carotenoids, tocopherols, and the phytohormone abscisic acid (ABA). The three rate-limiting enzymes CLA1 (At4g15560), PSY (At5g17230), and NCED3 (At3g14440) are indicated next to the corresponding arrows. The pathways are color-coded to match the circadian expression profiles for genes involved in the synthesis of **(b) **GGDP, **(c) **tocopherols, **(d) **carotenoids, and **(e) **ABA. Large colored arrows in panel a represent steps carried out by enzymes encoded by circadian-regulated genes (shown as thick lines in panels b to e). Medium-sized colored arrows in panel a represent a gene determined to be rhythmically expressed based on visual inspection, but that does not pass the stringent cut-off for being considered circadian regulated (pMMC-β < 0.05; shown as thin line in panel d). Thin black arrows shown in panel a represent genes that do not appear to be circadian regulated. Dashed arrows in panel a and dashed data series in panels b to d represent circadian genes that do not match the consolidated phase of expression of the other circadian genes in the pathways. The dashed data series in panel d corresponds to *NPQ1 *(At1g08550), which is the gene responsible for the conversion of violaxanthin back to zeaxanthin (shown as dashed arrow in panel a). The dashed line in panel b corresponds to *IPP1 *(At5g16440) and that in panel c corresponds to *VTE2 *(At2g18950). Panel e shows the mean circadian expression profiles of genes that are both circadian regulated and ABA induced (black; *n* = 492) and circadian-regulated ABA biosynthetic genes (green). The data shown in panels b to e are from the combined Covington plus Edwards dataset CCEE. Expression levels are plotted on the y-axes and time in constant light is plotted on the x-axes. For panels b to e, white and gray shading represent subjective day and night, respectively.

As in other studies, we identified an enrichment of clock regulation among genes functioning in many metabolic and physiological pathways [4-8]. We now report that genes implicated in the synthesis of geranylgeranyl diphosphate (GGDP) have a higher incidence of clock regulation than expected by chance. GGDP is a metabolite that is important in both primary and secondary metabolism, leading to the production of a variety of isoprenoids such as chlorophylls, carotenoids, tocopherols, and the phytohormones abscisic acid (ABA) and gibberellic acid (GA). These compounds are important for photosynthesis and dealing with oxidative stress, as well as for plant growth, development, and other stress responses [41-45]. GGDP synthesis occurs in the plastids via the methyl erythritol phosphate (MEP) pathway (Figure 5a). Six of the genes that are involved in the synthesis of GGDP from pyruvate and D-glyceraldehyde-3-phosphate are clock regulated (6/18 [33.3%]); five of these reach peak transcript levels during the subjective morning (Figure 5b), including *CLA1 *(*CLOROPLASTOS ALTERADOS 1*), which encodes the enzyme that carries out the first and rate-limiting step of the MEP pathway [46]. It has been shown that emission of a simple volatile product of this pathway, isoprene, is circadian regulated in oil palm and poplar [47,48]. Because the accumulation of chlorophylls, carotenoids, tocopherols, ABA, and GA is limited by MEP pathway activity [46], the extensive clock regulation of these biosynthetic genes probably has consequences for multiple aspects of plant physiology.

Many genes that encode enzymes acting downstream of the MEP pathway in the biosynthesis of complex isoprenoids are themselves clock regulated. More than 85% (7/8; *P *value for circadian enrichment = 1.7 × 10^-3^) of the genes involved in the conversion of GGDP and tyrosine into the various tocopherols and tocotrienols that together comprise the antioxidant vitamin E are clock regulated, six with a morning phase of peak transcript abundance (Figure 5c). Furthermore, genes encoding enzymes that act several steps upstream of tyrosine synthesis are also circadian regulated with the same morning phase (data not shown).

Similarly, we found a strikingly significant enrichment (10/12 [83%]; *P *= 3.1 × 10^-4^) of circadian regulation among genes encoding enzymes that are involved in the synthesis of carotenoids from GGDP, with most showing a peak phase of transcript abundance at around subjective dawn (Figure 5d). Notably, the transcript abundance of *PSY *(*PHYTOENE SYNTHASE*), encoding the first and rate-limiting enzyme in carotenoid biosynthesis [49], is clock controlled (Figure 5d). Carotenoids play an essential role in the process of nonphotochemical quenching, which allows plants to quench excited chlorophyll and prevent oxidative damage under excessive light conditions. In contrast to the dawn-phased transcript accumulation of carotenoid biosynthetic genes, *NPQ1 *(a gene encoding violaxanthin deepoxidase) has peak transcript levels at subjective dusk (Figure 5d). Violaxanthin deepoxidase acts antagonistically to the other clock-regulated carotenoid biosynthetic genes by recycling the carotenoid violaxanthin into compounds upstream of violaxanthin synthesis as part of the nonphotochemical quenching process [50]. Therefore, the antagonistic function of *NPQ1 *coincides well with its antiphasic transcript accumulation pattern to other clock-regulated carotenoid genes.

Carotenoids are also precursors to the hormone ABA, and over-expression of either *CLA1 *or *PSY *results in increased levels of carotenoids and ABA [46,49]. Additionally, the transcripts of the clock-regulated ABA metabolic genes *NCED3 *(*NINE-CIS-EPOXYCAROTENOID DIOXYGENASE*) and *ABA2 *(*ABA DEFICIENT 2*) accumulate during the subjective morning (Figure 5e). *NCED3 *encodes the rate-limiting activity for ABA biosynthesis [51]. The extensive clock regulation of genes implicated in ABA synthesis led us to examine whether ABA-responsive genes might also be enriched for circadian regulation.

### Extensive circadian regulation of hormone-responsive genes

**Figure 6:**
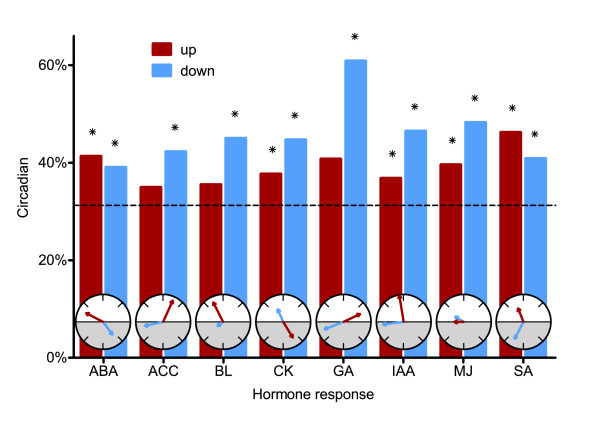
Hormone-responsive genes are circadian regulated. The proportions of clock-regulated genes among all that are upregulated or downregulated by each phytohormone are plotted as columns. Asterisks indicate statistically significant circadian enrichment (*P *< 0.05). The overlaid polar plots show the average circadian phases of expression for the hormone-responsive genes. The white and shaded portions of each polar plot represent subjective day and night, respectively, with subjective dawn at the left and subjective dusk at the right. The longer the arrow, the greater the degree of phase consolidation for each group of circadian-regulated genes.

ABA levels have previously been shown to fluctuate with diurnal rhythms in multiple plant species [52-55]. In addition, a significant overlap was recently reported between genes induced either by ABA or methyl jasmonate and genes that oscillate in light/dark cycles [56] (Table 2). However, because the transcript abundance of virtually all *Arabidopsis *genes is rhythmic in response to environmental cues [8], processes that exhibit diurnal regulation are not necessarily clock regulated. To search for a link between the circadian clock and ABA signaling, we looked for overlap between clock-regulated and ABA-induced [57] genes. More than 40% of ABA-induced genes (492/1,194) are circadian regulated, representing a significant enrichment (*P *= 2.7 × 10^-14^; Figure 6). The majority of these genes reach peak transcript levels during the subjective morning (Figure 5e) with a phase distribution significantly different from that of all circadian-regulated genes together (χ^2 ^test; *P *= 8.0 × 10^-23^). This morning phase distribution coincides with the phase of accumulation of *CLA1*, *PSY*, *NCED3*, and other circadian-regulated transcripts that are involved in the production of the ABA precursor violaxanthin or ABA itself (Figure 5e). These data suggest that ABA levels are clock regulated, indirectly leading to circadian cycling of ABA-responsive genes.

**Table 2:** Circadian-enriched hormone and stress response pathways

Treatment	% Circadian (circadian/expressed [*n*])	*P *value for over-representation	Other reports of enrichment of genes with	
			
			Diurnal regulation	Circadian regulation
Abscisic acid (up)	41% (492/1194)	2.7 × 10^-14^	[56]	-
Abscisic acid (down)	39% (500/1282)	5.5 × 10^-10^	[56]	-
1-Aminocyclopropane-1-carboxylic acid (up)	35% (36/103)	0.25	-	-
1-Aminocyclopropane-1-carboxylic acid (down)	42% (139/329)	1.6 × 10^-05^	-	-
Brassinolide (up)	35% (75/213)	0.13	-	[62]
Brassinolide (down)	45% (153/340)	6.2 × 10^-08^	-	-
Cytokinin (up)	38% (97/257)	1.6 × 10^-02^	-	-
Cytokinin (down)	45% (59/132)	8.3 × 10^-04^	-	-
Gibberellic acid (up)	39% (10/26)	0.28	-	-
Gibberellic acid (down)	61% (42/69)	3.9 × 10^-07^	-	-
Indole-3-acetic acid (up)	37% (108/295)	2.9 × 10^-02^	-	[7]
Indole-3-acetic acid (down)	47% (139/299)	2.2 × 10^-08^	-	-
Methyl jasmonate (up)	40% (242/611)	5.9 × 10^-06^	[56]	-
Methyl jasmonate (down)	48% (303/628)	9.9 × 10^-20^	[56]	-
Salicylic acid (up)	46% (153/331)	7.0 × 10^-09^	-	-
Salicylic acid (down)	41% (94/230)	1.3 × 10^-03^	-	-
Cold	41% (46/111)	1.5 × 10^-02^	-	[78]
Heat	53% (30/57)	6.6 × 10^-04^	-	-
Osmoticum	49% (18/37)	2.0 × 10^-02^	-	[78]
Salt	50% (62/124)	1.1 × 10^-05^	-	[78]
Water deprivation	51% (36/70)	3.6 × 10^-04^	-	-

In addition to diurnal changes in ABA abundance, it has been reported that other hormones such as auxins, brassinosteroids, cytokinins, ethylene, and gibberellins fluctuate over day/night cycles [52-55,58-61]. Furthermore, there is a significant overlap between brassinolide-induced and clock-regulated genes [62]. To investigate further the connections between the circadian clock and hormone signaling, we systematically examined genes that respond to these or other hormones within 30 minutes to 4 hours after treatment [57,63]. Strikingly, for every plant hormone analyzed there is a significant enrichment of circadian-regulated hormone-responsive genes. Specifically, we found circadian enrichments for genes that are induced in response to ABA, cytokinin, indole-3-acetic acid (IAA), methyl jasmonate (MJ), or salicylic acid (SA), as well as for genes downregulated in response to ABA, 1-aminocyclopropane-1-carboxylic acid (ACC; a key intermediate in ethylene biosynthesis), brassinolide, cytokinin, GA, IAA, MJ, or SA (Figure 6 and Table 2). Although changes in transcript abundance do not always correlate with changes in the abundance or activity of the corresponding protein [64,65], circadian changes in transcript levels of hormone-regulated genes probably indicates changes in either hormone levels or signaling pathway activity. Thus, our data suggest that the circadian clock modulates all of these hormone signaling pathways, perhaps helping to explain the pervasive effects of the clock on plant growth and development [66].

### Possible links between the clock and hormone signaling

The gaseous hormone ethylene plays well-known roles in fruit ripening and the triple response during seedling emergence; in addition, it is involved in organ senescence and abscission and responses to both abiotic and biotic stresses [67]. Production of ethylene has long been recognized as robustly clock regulated [68-70], but the mechanism linking the clock to rhythmic ethylene production is not currently understood. *ACS8 *(*ACC SYNTHASE 8*; At4g37770), a gene that is involved in the production of ethylene, has previously been shown to be circadian regulated with peak accumulation during the subjective day, the same time as peak ethylene emission; however, plants with a T-DNA insertion within the *ACS8 *coding region do not exhibit altered ethylene rhythms [69]. Under typical conditions, ACC synthase is believed to be the rate-limiting step of ACC biosynthesis. Under certain circumstances, however, ACC oxidase becomes the rate-limiting step [71]. Intriguingly, we found two genes that encode putative ACC oxidase enzymes (At1g04350 and At5g63600) are circadian regulated, with a similar phase of transcript accumulation as *ACS8 *(data not shown). It is possible that all three enzymes act together to generate circadian ethylene emission.

**Figure 7:**
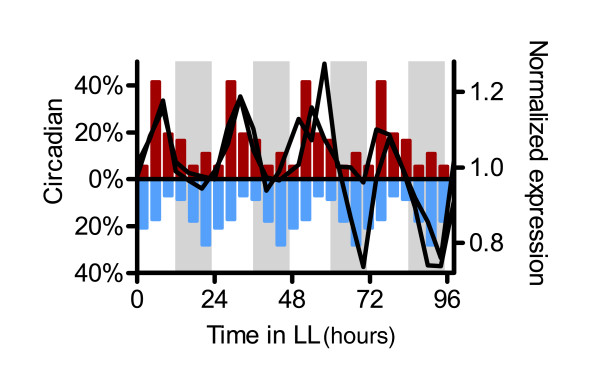
Co-expression of hormone-induced genes with signaling genes. Circadian phase distributions of 1-aminocyclopropane-1-carboxylic acid (ACC)- induced (red, above x-axis) and ACC-repressed (blue, below x-axis) genes are shown as histograms quadruple plotted on the left y-axes. Time series data are shown for *EIN3 *(At3g20770) and *EIL1 *(At2g27050), circadian-regulated genes involved in ACC signalling (black). Expression levels from the combined Covington plus Edwards dataset CCEE are plotted on the right y-axis and time in constant light is plotted on the x-axis. White and gray shading represent subjective day and night, respectively.

We next examined the relationship between the circadian phases of peak transcript abundance of ethylene signaling and ethylene responsive genes. Interestingly, two key ethylene signaling components, namely *EIN3 *(*ETHYLENE INSENSITVE 3*) and *EIL1 *(*EIN3-LIKE 1*), have a similar day-phased pattern of transcript accumulation as the ACC-induced genes (Figures 6 and 7). Conversely, the ACC-repressed genes tend to exhibit peak transcript abundance at times when the ACC signaling transcripts are at trough levels (Figures 6 and 7). It has been proposed that EIN3 and EIL1 mediate the majority of ethylene responses during seedling growth [72]. Notably, levels of *EIN3 *and *EIL1 *expression are not regulated by ethylene, indicating that the circadian clock regulates these transcripts independently of clock regulation of ethylene production [73,74]. Our findings suggest that the clock-regulated transcript abundance of ACC-induced genes may be due to a combination of circadian ethylene production and circadian-regulation of signaling components; further studies are needed to determine the relative contributions.

### Circadian regulation of abiotic stress responses

Multiple plant hormones have been implicated in stress responses [67,75-77] and many acute abiotic stresses are the direct result of daily light/dark cycles. As such, genes that are involved in perception, signaling and/or responses related to environmental stresses might be expected to be under clock control. Indeed, circadian regulation of salt-, osmoticum-, and cold-regulated genes has previously been demonstrated [4,78] (Table 2). By analyzing circadian fluctuations in transcript levels from genes grouped by Gene Ontology term, we identified additional stress-response pathways that are likely to be influenced by the clock, suggesting that the circadian clock is implicated not only in plant responses to cold, salt and drought, but also in responses to heat and reactive oxygen species (ROS).

**Figure 8:**
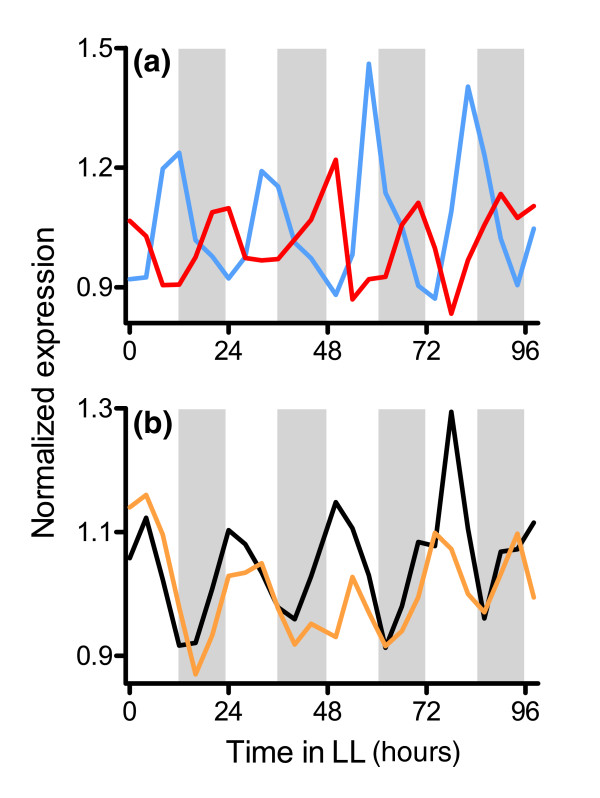
Stress-responsive genes are circadian regulated. **(a) **Circadian-regulated heat-induced genes are expressed before subjective dawn, completely out of phase with cold-induced genes. The average expression profile of heat-induced genes is indicated in red (*n* = 30), whereas that of cold-induced genes is indicated in blue (n = 46). **(b) **Circadian-regulated genes responsive to the reactive oxygen species hydrogen peroxide or to oxidative damage are expressed during the early subjective day. The average expression profile of genes induced by these compounds is shown in black (*n *= 41); for comparison, the average expression profile of genes involved in the light-harvesting reactions of photosynthesis is shown in orange (*n* = 60). The data shown are from the combined Covington plus Edwards data set CCEE. Mean expression levels are plotted on the y-axes and time in constant light is plotted on the x-axes. White and gray shading represent subjective day and night, respectively.

Genes that are classified as heat responsive have a significantly higher degree of circadian-regulation (53% [30/57]; *P *= 6.6 × 10^-4^) than do cold-responsive genes (41% [46/111]; *P *= 1.5 × 10^-2^). The average circadian transcript abundance profile of heat-responsive genes peaks just before subjective dawn, whereas cold-responsive genes reach peak transcript levels 12 hours later, near subjective dusk (Figure 8a). Such regulation may contribute to the competitive advantage provided by the circadian clock. Indeed, a circadian rhythm in heat resistance has been reported for cotton seedlings [79]. Strikingly, in this study seedlings were very resistant to extreme heat when it was applied near subjective dawn but the chances of survival plummeted to nil if heat exposure occurred around subjective dusk [79]. Plants are therefore most tolerant to heat treatment at the time of peak accumulation of heat-induced transcripts. A similar pattern is seen for cold tolerance; survival is optimal when plants are cold treated near to subjective dusk, when cold-regulated genes exhibit peak transcript abundance [80]. Our finding that one-half of heat responsive genes are also clock-regulated lays the foundation for future studies determining the mechanism of rhythmic heat stress resistance.

As well as generating predictable changes in temperature, the earth's daily rotation causes rhythms in light availability. Although light is essential for photosynthesis and plant survival, excess light leads to the accumulation of ROS that can damage the photosynthetic machinery and the plant [81]. ROS production is even more pronounced under stress conditions such as bright light, drought, or extreme temperatures [82]. Because genes that are involved in the synthesis of the compounds (carotenoids and tocopherols) that prevent ROS production through nonphotochemical quenching are clock regulated, with transcript levels peaking near subjective dawn (Figure 5c-d), it is interesting that 34% (41/122) of genes induced by ROS or oxidative damage are also clock-regulated. Although this is not a statistically significant enrichment, the average transcript profile for these genes peaks early in the subjective day, with a phase similar to that of genes involved in the light-harvesting reactions of photosynthesis (Figure 8b). It may be that clock regulation of photosynthetic and ROS responsive genes helps plants optimize photosynthetic activity while minimizing cellular damage caused by this process.

Abiotic stress responses appear to be highly interconnected, perhaps because related stresses often occur concurrently. Signaling pathways for stress-related hormones such as ABA, SA, MJ, and ethylene are believed to be important components in the crosstalk between stress signaling pathways [83]. The high degree of circadian regulation among genes responsive to various hormones and stresses might lead one to predict that the same clock-controlled genes are regulated by many different abiotic stimuli. However, this is not the case; most circadian-regulated genes are regulated by only one or two different stresses or hormones. This is reminiscent of the limited overlap between hormone-responsive genes in general; multiple hormones may regulate the expression of a family of genes with similar functions, but each individual gene is seldom controlled by more than one or two hormones [57]. This pathway specificity may allow the plant to fine-tune responses for a variety of stress conditions. For example, the gene response profile of plants subjected to drought and heat stress together is very different than the union of genes regulated by heat or drought alone [84].

## Conclusion

Our analysis of several circadian microarray experiments suggests that between 30% and 40% of expressed genes are clock regulated in seedlings. Transcript profiling and bioinformatic analyses are leading to a better understanding of the *cis *and *trans *factors that control these rhythmic changes in transcript abundance; in particular, bioinformatic analysis of promoter sequences has implicated several discrete motifs in phase-specific regulation of clock-controlled genes. Examination of pathways with an over-representation of clock-regulated genes is giving us insight into new aspects of plants physiology influenced by the clock. Of special interest is the extensive circadian regulation of all of the hormone and many of the environmental stress signaling pathways that we have examined. These new findings suggest most aspects of plant physiology are influenced by the circadian system and will help to lead us to a mechanistic understanding of how clocks provide an adaptive advantage.

## Materials and methods

### Verification of rhythmic expression by RT-PCR

The gene selection procedure involved randomly choosing genes with varying degrees of robust rhythmic expression. We chose three genes from the top third highest amplitude cyclers (At1g06460: 5'-CAT CTC TCG TCC CCT TGA AC-3' and 5'-AGG CCT TTC CTT TTG CAG AT-3'; At1g69830: 5'-CCC AGT TTC TTC GTC CTT CA-3' and 5'-CAA AAG TCA ATC GCG GAA AT-3'; and At5g12110: 5'-ATC TCC ACA CAG AGC GAG GT-3' and 5'-GCA GCT TCT CTC TCT TCA GCA-3') and three from the lowest third amplitude cyclers (At3g22970: 5'-GCC ATT TAC GAT GAA GAT CCA-3' and 5'-CGT CGG CTA ACA GAT TCC TC-3'; At1g45688: 5'-AAT CAC CAT CAC GCG ACT CT-3' and 5'-CAG CTT GGA TCT TAA GCG TCT-3'; and At3g04760: 5'-TCA GGC TGT CCG AAT TTC TCG AGA-3' and 5'-CCT CTG AAC TCG TTG GTT TCA CTA TCC-3'). For each time point, circadian transcript levels were normalized by dividing by transcript levels of the control gene *UBQ10 *(which encodes polyubiquitin 10; At4g05320: 5'-TCA AAT CTC TCT ACC GTG ATC AAG-3' and 5'- TTA CAT GAA ACG AAA CAT TGA ACT TC-3'). Semi-quantitative PCR was conducted as previously described [85].

### Comparison of circadian microarray datasets

The Harmer dataset was composed of technical replicates using Affymetrix *Arabidopsis *Genome Arrays (Affymetrix Inc., Santa Clara, CA, USA) [4]. We randomly assigned these replicates into separate unreplicated sets 20 different times. These were reanalyzed side-by-side with the Covington dataset (Affymetrix *Arabidopsis *ATH1 Genome Array) [7]. Because different sets of genes are represented on the two microarray platforms, we focused on genes common to both arrays that are also expressed in each dataset. We defined a gene as expressed if the Affymetrix MAS5.0 software called it 'Present' in at least four out of 12 samples (or out of the first 12 of 13 samples for the Edwards dataset).

Both the Edwards and Covington datasets were originally analyzed with the same circadian detection algorithm, namely COSOPT. However, the Edwards analysis did not use the initial sampling density weighted linear regression detrending, resulting in an increased number of genes identified as circadian [6]. To compare the extent of circadian regulation of genes expressed in both datasets, we reanalyzed the Covington dataset using the Edwards protocol, ignoring the dChip-derived standard error value and omitting the detrending step. Similarly, we analyzed the Michael datasets using the COSOPT parameters originally reported by Edwards and coworkers [6]. The Edwards and Covington datasets were combined in three different ways (as described under Results and discussion, above), and then analyzed using COSOPT [16]. Only genes defined as expressed in both individual datasets were considered expressed in the combined dataset.

### Genome organization of circadian-regulated genes

Groups of adjacent expressed genes in a sliding window (of sizes two, five, and ten genes) were evaluated based on the proportion displaying circadian expression patterns, the mean pMMC-β value (a measure of circadian rhythmicity), or the mean combinatorial pair-wise Pearson correlation coefficient. Threshold values were empirically derived via an approach based on a method originally proposed for quantitative trait mapping [86]. Specifically, we calculated the strongest cluster score for each of 1,000 random permutations of the data. From these values, we used the 95th percentile as an estimated experiment-wise critical value to detect circadian clusters in the genome with an overall type I error rate less than 5%. For the first two approaches, statistically significant local clusters of circadian-regulated genes were only detected when we grouped genes by phase of peak transcript abundance (using bins either 2 hours or 4 hours wide). This analysis was performed using scripts written in the statistical programming language R [87].

### Analysis of circadian clock regulatory elements

We employed four different strategies to identify potential motifs of interest: a trio of established motif discovery tools (stand-alone versions of AlignACE v2004 [88,89], Weeder v1.2 [90,91], and MotifSampler v3.2 [92,93]) and an exhaustive *in silico *testing of 6-mer and 8-mer nucleotide sequences.

The following validation protocol using both the Covington and Edwards datasets helped to narrow the list of putative CCREs to a more tractable size (from 55,107 to 126). For both the Covington and Edwards datasets, a potential motif must be over-represented in circadian genes versus all expressed genes; over-represented in at least one phase-specific subset of circadian genes versus all circadian genes; and under-represented in at least one phase-specific subset of circadian genes vs. all circadian genes. Over-representation and under-representation was determined using a previously described permutation testing approach [7,94]. Subsequent clustering of motifs based solely on sequence similarity (as measured using an scoring approach based on that used for Clustal [95]) enabled us to reduce further the number of motifs of interest by consolidating sequences with slight variations. These analyses were performed using scripts written in Perl and the statistical programming language R [87].

### Determination of pathway over-representation

Using annotations for the circadian-regulated genes found in the C+E intersection (see Additional data file 2), we searched for functionally-related gene groups enriched for circadian patterns of transcript accumulation. Genes were grouped according to annotations based on MapMan bins [96], Gene Ontology terms [25], and The *Arabidopsis *Information Resource [97] gene families, as well as information gleaned from the primary literature. Over-representation of circadian-regulated genes was determined using Fisher's exact test.

## Abbreviations

ABA: abscisic acid; ACC: 1-aminocyclopropane-1-carboxylic acid; CBS: CCA1-binding site; CCA1: circadian clock associated 1; CCRE: circadian clock regulatory element; EE: evening element; GA: gibberellic acid; GGDP: geranylgeranyl diphosphate; ME: morning element; MEP: methyl erythritol phosphate; MJ: methyl jasmonate; PBX: protein box element; ROS: reactive oxygen species; RT-PCR: reverse transcription polymerase chain reaction; SA: salicylic acid; TCP: teosinte branched1/cycloidia/PCF; TOC1: timing of CAB expression 1.

## Authors' contributions

MFC, SAK, and SLH developed the experimental design. MFC conducted the experiments. MFC, SLH, JNM, and MS analyzed the data. MFC and SLH drafted the manuscript. All authors read and approved the final manuscript.

## Additional data files

The following additional data are available with the online version of this paper. Additional data file 1 is a table listing the normalized circadian expression data for the combined Covington and Edwards dataset CCEE. Additional data file 2 is a table summarizing the expressed and circadian genes identified using different circadian microarray datasets.

## Supplementary Material

Additional data file 1 A table listing the normalized circadian expression data for the combined Covington and Edwards dataset CCEE. Click here for file

Additional data file 2 A table summarizing the expressed and circadian genes identified using different circadian microarray datasets. Click here for file

## References

[B1] Gardner MJ, Hubbard KE, Hotta CT, Dodd AN, Webb AA (2006). How plants tell the time.. Biochemical J.

[B2] Dodd AN, Salathia N, Hall A, Kevei E, Toth R, Nagy F, Hibberd JM, Millar AJ, Webb AA (2005). Plant circadian clocks increase photosynthesis, growth, survival, and competitive advantage.. Science.

[B3] Woelfle MA, Ouyang Y, Phanvijhitsiri K, Johnson CH (2004). The adaptive value of circadian clocks: an experimental assessment in cyanobacteria.. Curr Biol.

[B4] Harmer SL, Hogenesch JB, Straume M, Chang HS, Han B, Zhu T, Wang X, Kreps JA, Kay SA (2000). Orchestrated transcription of key pathways in Arabidopsis by the circadian clock.. Science.

[B5] Schaffer R, Landgraf J, Accerbi M, Simon V, Larson M, Wisman E (2001). Microarray analysis of diurnal and circadian-regulated genes in *Arabidopsis*.. Plant Cell.

[B6] Edwards KD, Anderson PE, Hall A, Salathia NS, Locke JC, Lynn JR, Straume M, Smith JQ, Millar AJ (2006). FLOWERING LOCUS C mediates natural variation in the high-temperature response of the *Arabidopsis *circadian clock.. Plant Cell.

[B7] Covington MF, Harmer SL (2007). The circadian clock regulates auxin signaling and responses in *Arabidopsis*.. PLoS Biol.

[B8] Michael TP, Mockler TC, Breton G, McEntee C, Byer A, Trout JD, Hazen SP, Shen R, Priest HD, Sullivan CM, Givan SA, Yanovsky M, Hong F, Kay SA, Chory J (2008). Network discovery pipeline elucidates conserved time-of-day-specific cis-regulatory modules.. PLoS Genet.

[B9] Emery P, Reppert SM (2004). A rhythmic Ror.. Neuron.

[B10] McClung CR (2006). Plant circadian rhythms.. Plant Cell.

[B11] Dunlap JC, Loros JJ (2004). The Neurospora circadian system.. J Biol Rhythms.

[B12] Alabadi D, Oyama T, Yanovsky MJ, Harmon FG, Mas P, Kay SA (2001). Reciprocal regulation between TOC1 and LHY/CCA1 within the *Arabidopsis *circadian clock.. Science.

[B13] Perales M, Mas P (2007). A functional link between rhythmic changes in chromatin structure and the *Arabidopsis *biological clock.. Plant Cell.

[B14] Harmer SL, Kay S (2005). Positive and negative factors confer phase-specific circadian regulation of transcription in *Arabidopsis*.. Plant Cell.

[B15] Hudson ME, Quail PH (2003). Identification of promoter motifs involved in the network of phytochrome A-regulated gene expression by combined analysis of genomic sequence and microarray data.. Plant Physiol.

[B16] Straume M (2004). DNA microarray time series analysis: automated statistical assessment of circadian rhythms in gene expression patterning.. Methods Enzymol.

[B17] Keegan KP, Pradhan S, Wang JP, Allada R (2007). Meta-analysis of *Drosophila *circadian microarray studies identifies a novel set of rhythmically expressed genes.. PLoS Comput Biol.

[B18] Michael TP, McClung CR (2003). Enhancer trapping reveals widespread circadian clock transcriptional control in *Arabidopsis*.. Plant Physiol.

[B19] Ren XY, Fiers MW, Stiekema WJ, Nap JP (2005). Local coexpression domains of two to four genes in the genome of *Arabidopsis*.. Plant Physiol.

[B20] Williams EJ, Bowles DJ (2004). Coexpression of neighboring genes in the genome of *Arabidopsis thaliana*.. Genome Res.

[B21] Lercher MJ, Urrutia AO, Hurst LD (2002). Clustering of housekeeping genes provides a unified model of gene order in the human genome.. Nat Genet.

[B22] Cohen BA, Mitra RD, Hughes JD, Church GM (2000). A computational analysis of whole-genome expression data reveals chromosomal domains of gene expression.. Nat Genet.

[B23] Michael TP, McClung CR (2002). Phase-specific circadian clock regulatory elements in *Arabidopsis*.. Plant Physiol.

[B24] Wang ZY, Kenigsbuch D, Sun L, Harel E, Ong MS, Tobin EM (1997). A Myb-related transcription factor is involved in the phytochrome regulation of an *Arabidopsis *Lhcb gene.. Plant Cell.

[B25] Ashburner M, Ball CA, Blake JA, Botstein D, Butler H, Cherry JM, Davis AP, Dolinski K, Dwight SS, Eppig JT, Harris MA, Hill DP, Issel-Tarver L, Kasarskis A, Lewis S, Matese JC, Richardson JE, Ringwald M, Rubin GM, Sherlock G (2000). Gene ontology: tool for the unification of biology. The Gene Ontology Consortium.. Nat Genet.

[B26] Hanano S, Stracke R, Jakoby M, Merkle T, Domagalska MA, Weisshaar B, Davis SJ (2008). A systematic survey in *Arabidopsis thaliana *of transcription factors that modulate circadian parameters.. BMC genomics.

[B27] Imaizumi T, Kay SA (2006). Photoperiodic control of flowering: not only by coincidence.. Trends Plant Sci.

[B28] Cheng XF, Wang ZY (2005). Overexpression of COL9, a CONSTANS-LIKE gene, delays flowering by reducing expression of CO and FT in *Arabidopsis thaliana*.. Plant J.

[B29] Ledger S, Strayer C, Ashton F, Kay SA, Putterill J (2001). Analysis of the function of two circadian-regulated CONSTANS-LIKE genes.. Plant J.

[B30] Hazen SP, Schultz TF, Pruneda-Paz JL, Borevitz JO, Ecker JR, Kay SA (2005). LUX ARRHYTHMO encodes a Myb domain protein essential for circadian rhythms.. Proc Natl Acad Sci USA.

[B31] Kuno N, Moller SG, Shinomura T, Xu X, Chua NH, Furuya M (2003). The novel MYB protein EARLY-PHYTOCHROME-RESPONSIVE1 is a component of a slave circadian oscillator in *Arabidopsis*.. Plant Cell.

[B32] Zhang X, Chen Y, Wang ZY, Chen Z, Gu H, Qu LJ (2007). Constitutive expression of CIR1 (RVE2) affects several circadian-regulated processes and seed germination in *Arabidopsis*.. Plant J.

[B33] Tsuda K, Tsuji T, Hirose S, Yamazaki K (2004). Three *Arabidopsis *MBF1 homologs with distinct expression profiles play roles as transcriptional co-activators.. Plant Cell Physiol.

[B34] Kooiker M, Airoldi CA, Losa A, Manzotti PS, Finzi L, Kater MM, Colombo L (2005). BASIC PENTACYSTEINE1, a GA binding protein that induces conformational changes in the regulatory region of the homeotic *Arabidopsis *gene SEEDSTICK.. Plant Cell.

[B35] Ikeda A, Nishina PM, Naggert JK (2002). The tubby-like proteins, a family with roles in neuronal development and function.. J Cell Sci.

[B36] Lai CP, Lee CL, Chen PH, Wu SH, Yang CC, Shaw JF (2004). Molecular analyses of the *Arabidopsis *TUBBY-like protein gene family.. Plant Physiol.

[B37] Navaud O, Dabos P, Carnus E, Tremousaygue D, Herve C (2007). TCP transcription factors predate the emergence of land plants.. J Mol Evol.

[B38] Palatnik JF, Allen E, Wu X, Schommer C, Schwab R, Carrington JC, Weigel D (2003). Control of leaf morphogenesis by microRNAs.. Nature.

[B39] Kim WY, Fujiwara S, Suh SS, Kim J, Kim Y, Han L, David K, Putterill J, Nam HG, Somers DE (2007). ZEITLUPE is a circadian photoreceptor stabilized by GIGANTEA in blue light.. Nature.

[B40] Perales M, Portoles S, Mas P (2006). The proteasome-dependent degradation of CKB4 is regulated by the *Arabidopsis *biological clock.. Plant J.

[B41] Christmann A, Moes D, Himmelbach A, Yang Y, Tang Y, Grill E (2006). Integration of abscisic acid signalling into plant responses.. Plant Biol.

[B42] Fleet CM, Sun TP (2005). A DELLAcate balance: the role of gibberellin in plant morphogenesis.. Curr Opin Plant Biol.

[B43] Howitt CA, Pogson BJ (2006). Carotenoid accumulation and function in seeds and non-green tissues.. Plant Cell Environ.

[B44] Maeda H, DellaPenna D (2007). Tocopherol functions in photosynthetic organisms.. Curr Opin Plant Biol.

[B45] Jensen PE, Bassi R, Boekema EJ, Dekker JP, Jansson S, Leister D, Robinson C, Scheller HV (2007). Structure, function and regulation of plant photosystem I.. Biochim Biophys Acta.

[B46] Estevez JM, Cantero A, Reindl A, Reichler S, Leon P (2001). 1-Deoxy-D-xylulose-5-phosphate synthase, a limiting enzyme for plastidic isoprenoid biosynthesis in plants.. J Biol Chem.

[B47] Loivamaki M, Louis S, Cinege G, Zimmer I, Fischbach RJ, Schnitzler JP (2007). Circadian rhythms of isoprene biosynthesis in grey poplar leaves.. Plant Physiol.

[B48] Wilkinson MJ, Owen SM, Possell M, Hartwell J, Gould P, Hall A, Vickers C, Nicholas Hewitt C (2006). Circadian control of isoprene emissions from oil palm (*Elaeis guineensis*).. Plant J.

[B49] Lindgren LO, Stalberg KG, Hoglund AS (2003). Seed-specific overexpression of an endogenous *Arabidopsis *phytoene synthase gene results in delayed germination and increased levels of carotenoids, chlorophyll, and abscisic acid.. Plant Physiol.

[B50] DellaPenna D, Pogson BJ (2006). Vitamin synthesis in plants: tocopherols and carotenoids.. Annu Rev Plant Biol.

[B51] Tian L, DellaPenna D, Zeevaart JA (2004). Effect of hydroxylated carotenoid deficiency on ABA accumulation in *Arabidopsis*.. Physiologia Plantarum.

[B52] Burschka C, Tenhunen JD, Hartung W (1983). Diurnal variations in abscisic acid content and stomatal response to applied abscisic acid in leaves of irrigated and non-irrigated *Arbus unedo *plants under naturally fluctuating envirnomental conditions.. Oecologia (Berlin).

[B53] Cheikh N, Brenner ML (1992). Regulation of key enzymes of sucrose biosynthesis in soybean leaves: effect of dark and light conditions and role of gibberellins and abscisic acid.. Plant Physiol.

[B54] Novakova M, Motyka V, Dobrev PI, Malbeck J, Gaudinova A, Vankova R (2005). Diurnal variation of cytokinin, auxin and abscisic acid levels in tobacco leaves.. J Exp Bot.

[B55] Lee KH, Piao HL, Kim HY, Choi SM, Jiang F, Hartung W, Hwang I, Kwak JM, Lee IJ, Hwang I (2006). Activation of glucosidase via stress-induced polymerization rapidly increases active pools of abscisic acid.. Cell.

[B56] Mizuno T, Yamashino T (2008). Comparative transcriptome of diurnally oscillating genes and hormone-responsive genes in *Arabidopsis thaliana*: insight into circadian clock-controlled daily responses to common ambient stresses in plants.. Plant Cell Physiol.

[B57] Nemhauser JL, Hong F, Chory J (2006). Different plant hormones regulate similar processes through largely nonoverlapping transcriptional responses.. Cell.

[B58] Machackova I, Chauvaux N, Dewitte W, Van Onckelen H (1997). Diurnal fluctuations in ethylene formation in *Chenopodium rubrum*.. Plant Physiol.

[B59] Lee IJ, Foster KR, Morgan PW (1998). Photoperiod control of gibberellin levels and flowering in sorghum.. Plant Physiol.

[B60] Bancos S, Szatmari AM, Castle J, Kozma-Bognar L, Shibata K, Yokota T, Bishop GJ, Nagy F, Szekeres M (2006). Diurnal regulation of the brassinosteroid-biosynthetic CPD gene in *Arabidopsis*.. Plant Physiol.

[B61] Jouve L, Gaspar T, Kevers C, Greppin H, Degli Agosti R (1999). Involvement of indole-3-acetic acid in the circadian growth of the first internode of *Arabidopsis*.. Planta.

[B62] Dodd AN, Gardner MJ, Hotta CT, Hubbard KE, Dalchau N, Love J, Assie JM, Robertson FC, Jakobsen MK, Goncalves J, Sanders D, Webb AA (2007). The *Arabidopsis *circadian clock incorporates a cADPR-based feedback loop.. Science.

[B63] Krinke O, Ruelland E, Valentova O, Vergnolle C, Renou JP, Taconnat L, Flemr M, Burketova L, Zachowski A (2007). Phosphatidylinositol 4-kinase activation is an early response to salicylic acid in *Arabidopsis *suspension cells.. Plant Physiol.

[B64] Nozue K, Covington MF, Duek PD, Lorrain S, Fankhauser C, Harmer SL, Maloof JN (2007). Rhythmic growth explained by coincidence between internal and external cues.. Nature.

[B65] Gibon Y, Blaesing OE, Hannemann J, Carillo P, Hohne M, Hendriks JH, Palacios N, Cross J, Selbig J, Stitt M (2004). A robot-based platform to measure multiple enzyme activities in *Arabidopsis *using a set of cycling assays: comparison of changes of enzyme activities and transcript levels during diurnal cycles and in prolonged darkness.. Plant Cell.

[B66] Yakir E, Hilman D, Harir Y, Green RM (2007). Regulation of output from the plant circadian clock.. FEBS J.

[B67] van Loon LC, Geraats BP, Linthorst HJ (2006). Ethylene as a modulator of disease resistance in plants.. Trends Plant Sci.

[B68] Finlayson SA, Lee IJ, Morgan PW (1998). Phytochrome B and the regulation of circadian ethylene production in sorghum.. Plant Physiol.

[B69] Thain SC, Vandenbussche F, Laarhoven LJ, Dowson-Day MJ, Wang ZY, Tobin EM, Harren FJ, Millar AJ, Straeten D Van Der (2004). Circadian rhythms of ethylene emission in *Arabidopsis *.. Plant Physiol.

[B70] Rikin A, Chalutz E, Anderson JD (1984). Rhythmicity in ethylene production in cotton seedlings.. Plant Physiol.

[B71] Rieu I, Cristescu SM, Harren FJ, Huibers W, Voesenek LA, Mariani C, Vriezen WH (2005). RP-ACS1, a flooding-induced 1-aminocyclopropane-1-carboxylate synthase gene of Rumex palustris, is involved in rhythmic ethylene production.. J Exp Bot.

[B72] Guo H, Ecker JR (2004). The ethylene signaling pathway: new insights.. Curr Opin Plant Biol.

[B73] AtGenExpress Visualization Tool. http://jsp.weigelworld.org/expviz/expviz.jsp.

[B74] Zimmermann P, Hirsch-Hoffmann M, Hennig L, Gruissem W (2004). GENEVESTIGATOR. *Arabidopsis *microarray database and analysis toolbox.. Plant Physiol.

[B75] Halim VA, Vess A, Scheel D, Rosahl S (2006). The role of salicylic acid and jasmonic acid in pathogen defence.. Plant Biol.

[B76] Wasternack C (2007). Jasmonates: an update on biosynthesis, signal transduction and action in plant stress response, growth and development.. Ann Bot (London).

[B77] Seki M, Umezawa T, Urano K, Shinozaki K (2007). Regulatory metabolic networks in drought stress responses.. Curr Opin Plant Biol.

[B78] Kreps JA, Wu Y, Chang HS, Zhu T, Wang X, Harper JF (2002). Transcriptome changes for *Arabidopsis *in response to salt, osmotic, and cold stress.. Plant Physiol.

[B79] Rikin A (1992). Circadian rhythm of heat resistance in cotton seedlings: synthesis of heat-shock proteins.. Eur J Cell Biol.

[B80] Rikin A, Dillwith JW, Bergman DK (1993). Correlation between the circadian rhythm of resistance to extreme temperatures and changes in fatty acid composition in cotton seedlings.. Plant Physiol.

[B81] Foyer CH, Noctor G (2000). Oxygen processing in photosynthesis: regulation and signalling.. New Phytol.

[B82] Krieger-Liszkay A, Trebst A (2006). Tocopherol is the scavenger of singlet oxygen produced by the triplet states of chlorophyll in the PSII reaction centre.. J Exp Bot.

[B83] Fujita M, Fujita Y, Noutoshi Y, Takahashi F, Narusaka Y, Yamaguchi-Shinozaki K, Shinozaki K (2006). Crosstalk between abiotic and biotic stress responses: a current view from the points of convergence in the stress signaling networks.. Curr Opin Plant Biol.

[B84] Rizhsky L, Liang H, Shuman J, Shulaev V, Davletova S, Mittler R (2004). When defense pathways collide. The response of *Arabidopsis *to a combination of drought and heat stress.. Plant Physiol.

[B85] Martin-Tryon EL, Kreps JA, Harmer SL (2007). GIGANTEA acts in blue light signaling and has biochemically separable roles in circadian clock and flowering time regulation.. Plant Physiol.

[B86] Churchill GA, Doerge RW (1994). Empirical threshold values for quantitative trait mapping.. Genetics.

[B87] R Development Core Team (2007). R: a Language and Environment for Statistical Computing.

[B88] Hughes JD, Estep PW, Tavazoie S, Church GM (2000). Computational identification of cis-regulatory elements associated with groups of functionally related genes in *Saccharomyces cerevisiae*.. J Mol Biol.

[B89] Roth FP, Hughes JD, Estep PW, Church GM (1998). Finding DNA regulatory motifs within unaligned noncoding sequences clustered by whole-genome mRNA quantitation.. Nature Biotechnol.

[B90] Pavesi G, Mauri G, Pesole G (2001). An algorithm for finding signals of unknown length in DNA sequences.. Bioinformatics.

[B91] Pavesi G, Mereghetti P, Mauri G, Pesole G (2004). Weeder Web: discovery of transcription factor binding sites in a set of sequences from co-regulated genes.. Nucleic Acids Res.

[B92] Thijs G, Lescot M, Marchal K, Rombauts S, De Moor B, Rouze P, Moreau Y (2001). A higher-order background model improves the detection of promoter regulatory elements by Gibbs sampling.. Bioinformatics.

[B93] Thijs G, Marchal K, Lescot M, Rombauts S, De Moor B, Rouze P, Moreau Y (2002). A Gibbs sampling method to detect overrepresented motifs in the upstream regions of coexpressed genes.. J Comput Biol.

[B94] Walley J, Coughlan S, Hudson ME, Covington MF, Kaspi R, Banu G, Harmer SL, Dehesh K (2007). Mechanical stress induces biotic and abiotic stress responses via a novel cis-element.. PLoS Genet.

[B95] Chenna R, Sugawara H, Koike T, Lopez R, Gibson TJ, Higgins DG, Thompson JD (2003). Multiple sequence alignment with the Clustal series of programs.. Nucleic Acids Res.

[B96] Thimm O, Blasing O, Gibon Y, Nagel A, Meyer S, Kruger P, Selbig J, Muller LA, Rhee SY, Stitt M (2004). MAPMAN: a user-driven tool to display genomics data sets onto diagrams of metabolic pathways and other biological processes.. Plant J.

[B97] Swarbreck D, Wilks C, Lamesch P, Berardini TZ, Garcia-Hernandez M, Foerster H, Li D, Meyer T, Muller R, Ploetz L, Radenbaugh A, Singh S, Swing V, Tissier C, Zhang P, Huala E (2008). The *Arabidopsis *Information Resource (TAIR): gene structure and function annotation.. Nucleic Acids Res.

